# Establishing an Integrated Pest Management Program for Coffee Berry Borer (*Hypothenemus hampei*) in Hawaii and Puerto Rico Coffee Agroecosystems: Achievements and Challenges

**DOI:** 10.3390/insects14070603

**Published:** 2023-07-03

**Authors:** Luis F. Aristizábal, Melissa A. Johnson, Yobana A. Mariño, Paul Bayman, Mark G. Wright

**Affiliations:** 1 Department of Plant and Environmental Protection Sciences, College of Tropical Agriculture and Human Resources, University of Hawaii at Manoa, Honolulu, HI 96822, USA; luisaris@hawaii.edu (L.F.A.); markwrig@hawaii.edu (M.G.W.); 2 Daniel K. Inouye US Pacific Basin Agricultural Research Center, United States Department of Agriculture-Agricultural Research Service, Hilo, HI 96720, USA; 3 Department of Biology, University of Puerto Rico Rio Piedras, San Juan, PR 00931, USA; yobana.marino@upr.edu (Y.A.M.); bayman.upr@gmail.com (P.B.)

**Keywords:** *Coffea arabica*, biological control, cultural practices, monitoring, natural enemies

## Abstract

**Simple Summary:**

This review addresses research and extension efforts towards the development of an IPM program for coffee berry borer (CBB) in Hawaii and Puerto Rico. After more than a decade of living with CBB, some similarities and differences between these island regions can be seen. Although the benefits of monitoring fields for CBB activity were demonstrated, very few growers in either region currently use traps or 30-tree sampling to inform spray decisions. In Hawaii, growers may either visually inspect a small number of berries for CBB presence to inform spray times or are on a calendar spray system whereby fields are sprayed monthly regardless of infestation level. Farmers in Puerto Rico rarely conduct management for CBB, largely due to the compounding issues of other pests, diseases, and recurring damage from hurricanes. Subsidies for *Beauveria bassiana* are still available to growers in Hawaii, but only a small percentage of the total farms in the State take advantage of this program. At the core of these issues is a combination of an aging farmer population, a disconnect between farm owners and the issues that are occurring on their fields, and high costs that prohibit many growers from implementing the management that they know is needed but which they cannot afford.

**Abstract:**

Coffee berry borer (CBB) is the most serious insect pest of coffee worldwide, causing significant reductions in yield and quality. Following the introduction of CBB to Puerto Rico (2007) and Hawaii (2010), researchers, extension agents, industry representatives, and coffee growers have worked together to develop an integrated pest management (IPM) program that is both effective and economically feasible for each island. Since the introduction of the IPM program in Hawaii, research efforts have led to a better understanding of CBB population dynamics, as well as optimized monitoring, cultural practices, and commercial *Beauveria bassiana* applications. As a result of these efforts, a substantial reduction in average CBB infestation and an increase in coffee yields and quality have been documented in Hawaii over the last decade. However, significant challenges remain in addressing high production and labor costs, limited availability of labor, and a lack of training for field workers in both regions. Although considerable effort has gone into research to support CBB IPM in Hawaii and Puerto Rico, the adoption of these strategies by coffee farmers needs to be increased. More diversified methods of outreach and education are needed to reach growers in rural, isolated areas. Significant gaps exist in the ability and willingness of growers and workers to access and digest information online, emphasizing the importance of on-farm workshops and farmer-to-farmer teaching. Additional methods of training are needed to help coffee farmers and field workers learn how to properly conduct cultural controls and optimize the use of biological control agents such as *B. bassiana*.

## 1. Introduction

Within the United States, coffee is only commercially grown in the Hawaiian Islands (Pacific Ocean) and Puerto Rico (Caribbean). Both island regions have a reputation for producing high-quality coffee, which is currently planted on approximately 5000 ha in Puerto Rico [1] and 3800 ha in Hawaii [2,3]. The area cultivated with coffee on these islands is just a small fraction of the approximately 10 million ha planted globally [4], yet the coffee produced in these regions demands premium prices on the world specialty market due to its unique origin. In Hawaii, green coffee value was estimated at USD 113 M during the 2021–2022 coffee season [5]. In Puerto Rico, the estimated value of green coffee was USD 4.8M in 2018, an astonishing 86% decrease from six years earlier, mostly due to damage from Hurricanes Irma and Maria in 2017 [1].

*Coffea arabica* L. (Gentianales: Rubiaceae) has been grown in Hawaii and Puerto Rico for hundreds of years and has a rich cultural heritage in both regions [6,7,8]. However, smallholders, who represent most coffee farmers in both island regions, are facing major economic losses due to damage caused by invasive insect pests and diseases. Widely considered the most serious insect pest affecting coffee worldwide, the coffee berry borer (CBB, *Hypothenemus hampei* Ferrari) causes direct damage to the coffee seed (“bean”) resulting in reduced quality and yields [9,10,11,12]. Initially reported in Africa [13,14], CBB has spread to nearly all coffee-producing countries in tropical and subtropical regions around the world [4,15]. The entire CBB life cycle is completed inside the coffee berry, making it difficult to control with pesticides. The need for intensive year-round management of this pest has also led to increased production and labor costs, further reducing profits for growers. In the first five years following the invasion of CBB in Hawaii, it was estimated that coffee yields were reduced by an average of 20%, while the average price per pound of parchment coffee increased by 16% [16].

Several parallels between Hawaii and Puerto Rico allow for a comparison of these two island regions and their respective trajectories vis-à-vis the CBB. Given their isolation from nearby continental sources, these island regions were two of the last coffee-growing areas in the world to be invaded by this pest. CBB was not detected until 2007 in Puerto Rico [17] and 2010 in Hawaii [18]. As islands with considerable natural biodiversity and endemism, they suffer from (and are acutely aware of) an onslaught of biological invasions, with new plant pests and diseases detected each year. As US labor laws apply in both regions, the cost of labor is also much higher than in other coffee-producing countries [19]. Therefore, the manual collection of fruits during and after harvest, which is an important part of CBB management [19], is not always economically viable. EPA regulations also restrict the types of pesticides that can be used in these regions, with chemicals such as endosulfan and chlorpyrifos being prohibited due to their negative impacts on human and environmental health [20]. Finally, Hawaii and Puerto Rico have reported much higher levels of CBB infestation than other countries, sometimes reaching >85% [19,21], largely due to the higher costs and labor shortages that prevent many growers from implementing necessary interventions such as post-harvest sanitation. Given the similarities in geography, socioeconomics, and government regulations, collaborative research between Hawaii and Puerto Rico is timely and stands to provide valuable insights into the integrated pest management (IPM) of this invasive pest on a global scale.

**Figure 1:**
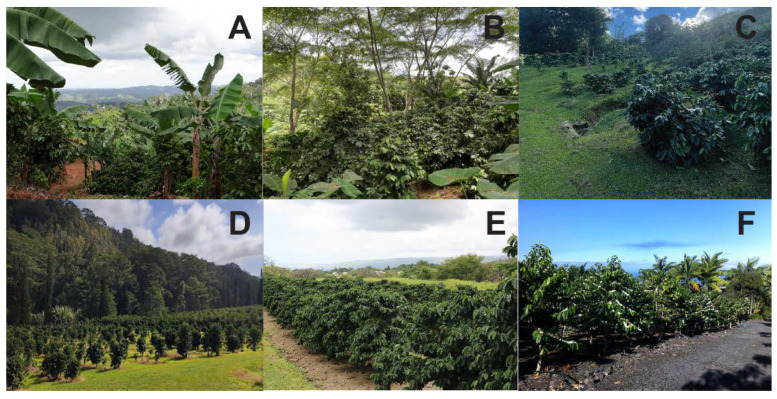
Coffee landscapes: (**A**–**C**) In Puerto Rico, arabica and robusta coffee are planted from 200–1000 m asl, with a few farms near sea level. Varieties such as caturra, catuai, and local selections of Limaní and Frontón grow on a mix of soil types, in full sun, or under tropical shade trees. (**D**–**F**) In Hawaii, cultivated varieties of *Coffea arabica*, including typica, bourbon, caturra, and catuai (among others), are planted from 200–900 m asl on gently sloping hills with deep soils in Ka’u (**D**,**E**) or steep rocky terrain in Kona (**F**), mostly in full sun. Photos: (**A**) P. Bayman; (**B**,**C**) Y. A. Mariño; (**D**) L. F. Aristizábal; (**E**,**F**) M. A. Johnson.

This review addresses efforts to establish a CBB IPM program that is economically feasible, environmentally sustainable, and has a high likelihood of being adopted by coffee growers. An effective CBB IPM program must not only be practical and reliable but also adapted to local conditions in Hawaiian and Puerto Rican coffee agroecosystems (Figure 1). Since the introduction of early IPM practices, several achievements have been made in key aspects of management, which have improved the quality of coffee grown in these regions. These achievements are due to basic and applied research and extension activities conducted with the participation of coffee growers through a five-year USDA area-wide grant; this review summarizes the studies and collaboration that resulted from this grant. We also discuss challenges that remain to be addressed in IPM development and implementation in both regions.

## 2. CBB Biology

**Figure 2:**
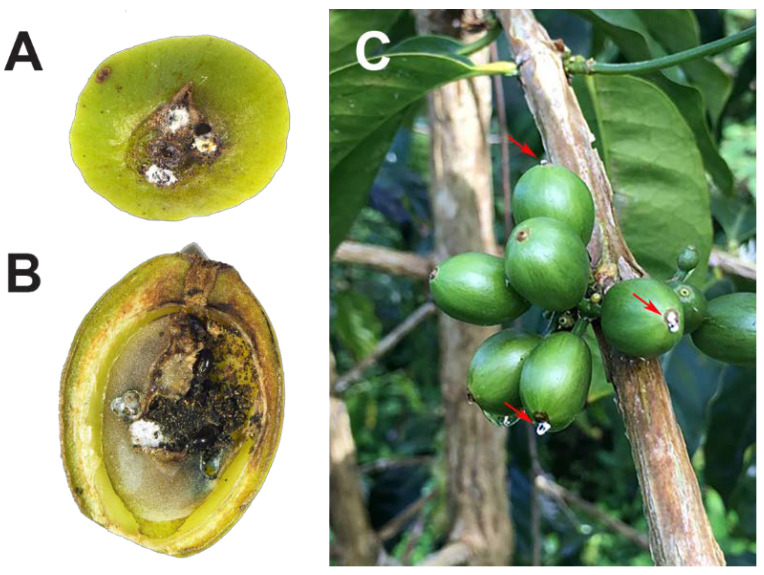
CBB females in the AB position killed by the entomopathogenic fungus *Beauveria bassiana* (**A**); CBB adult females in the CD position: note larval feeding on the seed tissue (**B**); red arrows show the white mycelia of *B. bassiana* growing from the bodies of the CBB in AB position (**C**). Photos: (**A**,**B**) M. A. Johnson; (**C**) L. F. Aristizábal.

Adult female CBB are attracted to developing berries and cherries that are 60–240 days old [10,22]. Coffee fruits are commonly called berries, and ripe fruits are called cherries, even though neither term is botanically correct; coffee fruits are drupes. Once an appropriate berry is located, the female bores a small entry hole, typically in the central disc area. The degree of penetration into the berry can be described by the AB and CD positions [9]. The AB position is the initial perforation of the exocarp and mesocarp, in which part of the CBB body is still visible (Figure 2A). CBB can persist in this AB position for 30–90 days until the endosperm (part of the coffee seed or “bean”) has reached <20% moisture content [10]. In the AB position, the CBB female is vulnerable to insecticides, pathogens, natural predators, and parasitoids and can be killed before damage to the endosperm occurs [9,23]. When the endosperm reaches > 20% dry weight (berries ~120–150 days old; [10]) the female will begin boring tunnels for reproduction. CBB entry into the endosperm and the building of galleries in which to initiate oviposition is known as the CD position (Figure 2B). In the CD position, damage to the coffee seed has occurred, and the CBB female and its progeny are protected from pesticides and the entomopathogenic fungus *Beauveria bassiana* (Figure 2C) [9,23]. Male and female siblings mate inside their natal berry, the males die, and the mated females fly in search of a new berry, thus starting a new cycle of infestation.

## 3. CBB Detection and Dispersal in Hawaii and Puerto Rico

In Puerto Rico, the first credible reports of CBB came from the central mountain range in the municipalities of San Sebastián and Utuado in 2007 [17]. Several earlier reports are considered spurious [24]. The first published, extensive census of CBB in Puerto Rico was conducted ten years later and found that it had spread throughout the island [21]. In Hawaii, CBB was first reported from the Kona district of Hawaii Island in 2010 [18] and continued to spread to the nearby coffee-growing districts of Ka’u, Puna, and Hamakua from 2011–2013 [16]. CBB was later detected on the neighboring islands of Oahu (2014), Maui (2016), Kauai, and Lanai (2020) [25]. CBB may have been brought to the islands in the clothing or equipment of migrant workers, through improperly fumigated shipments, or in coffee seeds brought in by shipments or air passengers [26,27]. A quantitative risk assessment model suggested that at least one air passenger per year could be bringing CBB-infested materials to Hawaii, highlighting the need for more stringent biosecurity measures at ports of entry to prevent the introduction of additional pests and diseases [28].

## 4. CBB Infestation, Damage, and Economic Implications

CBB populations and coffee infestation in Puerto Rico and Hawaii vary greatly in time and space, depending on several factors including plant phenology, weather, and management [29,30]. In a large-scale census conducted on 214 coffee farms across Puerto Rico in 2014, the average infestation was 20%, with a range of 1–95% [21]. Only 20% of the farms surveyed used *B. bassiana* (discussed below), and it was typically only applied once per season [21]. There was a significant positive correlation between the proportion of berries infested and the mean number of CBB per infested berry with increasing elevation [21]. This pattern was observed for both *C. arabica* and *C. canephora* (robusta coffee). Temperatures at elevations below 200 m in Puerto Rico are above the optimal range for CBB development (15–30 °C), which may explain why CBB is not as prevalent at the lowest elevations.

In contrast, trapping studies from Hawaii suggest greater numbers of CBB at low-elevation farms (200–400 m) compared to mid (401–600 m) and high (601–800 m) elevations [30]. This aligns with results from Hamilton et al. [31] who reported faster CBB development times at low elevations (and thus more generations per season) due to warmer temperatures. There may be other, more subtle environmental factors that impact CBB infestation. In some cases, adjacent fields with the same cultivar and similar management had very different levels of CBB infestation in Puerto Rico [21]. Infestation levels have been observed to vary greatly within a single field (M. Johnson, pers. obs.), likely due to differences in microclimate associated with vegetation and topological features, but appropriately scaled, detailed studies that correlate variation in CBB infestation with microclimate have not been published.

Management also has a significant impact on CBB populations and infestation. Johnson and Manoukis [32] reported that poorly managed farms on Hawaii Island had significantly higher numbers of CBB caught in traps compared to feral (wild) coffee and abandoned farms. CBB infestation was also significantly higher on poorly managed farms (63%) compared to well-managed, abandoned, and feral sites (<20%). The higher production on these poorly managed farms and lack of any management besides mowing/weed control provided CBB with easy access to berries, while feral and abandoned sites had few berries to infest, and these were difficult for CBB to access due to overgrown vegetation [32].

**Figure 3:**
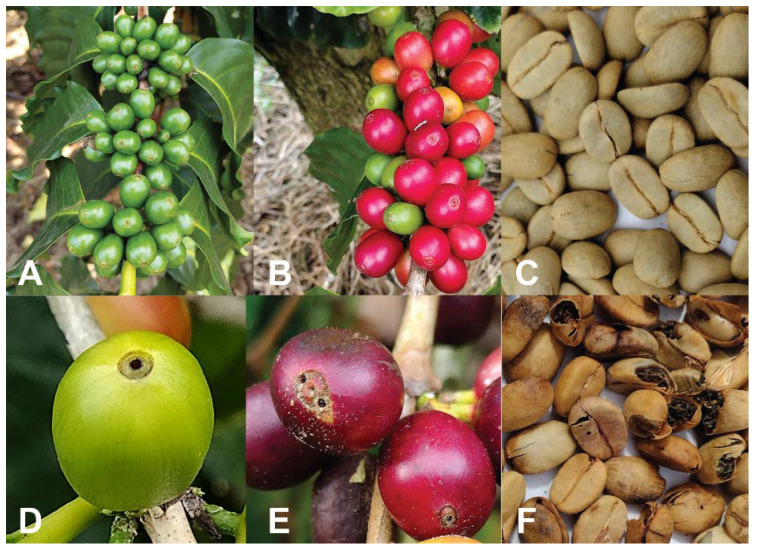
Healthy uninfested green berries (**A**), cherries (**B**), and processed parchment coffee (**C**). CBB infested green berries (**D**), cherries (**E**), and damaged parchment coffee (**F**). Photos: Luis F. Aristizábal.

High CBB infestation (>20% of berries infested) results in high losses in processed coffee. CBB entry holes, reproduction, and larval feeding in green beans reduce the quality and value of processed coffee (Figure 3). In extreme cases, 100% of the coffee yield may be lost [4,9,10]. In Hawaii, buyers of cherry or parchment (processed coffee) pay 10–15 cents less per pound than the standard price if CBB damage exceeds 10%. Prior to the CBB invasion in Hawaii, the highest quality of coffee (known as “Extra Fancy”) represented around 25% of the Kona crop. From 2011 to 2013, no coffee from Hawaii was certified as “Extra Fancy” or “Fancy”, with the best coffee certified for export being “Prime” (defect tolerance of 20% defective beans by weight), which is the second lowest grade according to Hawaiian standards of quality for coffee exportation [19]. In the first two years after CBB was detected, losses for the Hawaii coffee industry were estimated at USD 25.7M in sales, USD 12.7M in crop yields, and USD 7.6M in household profits [33]. In Puerto Rico, no studies have been published on the economic cost of CBB in terms of crop loss, decreased value of coffee due to a lower quality, or the cost of control measures. According to the former Puerto Rico Secretary of Agriculture, yield losses due to CBB are ~20–30%, though this varies from year to year (Carlos Flores, pers. comm.).

There is a long-running debate about where CBB survives in the inter-crop season in areas where coffee plant phenology is synchronous (which includes Puerto Rico and many areas of Hawaii). Johnson et al. [34] found that berries remaining on the trees after harvest serve as major reservoirs, as do fallen berries on the ground. While there were fewer berries remaining in the trees after the harvest, populations were larger, and mortality was lower relative to berries on the ground. In both areas, old berries (“raisins”) served as reservoirs for up to six months after the final harvest. In contrast, berries on the ground have been observed to decompose quickly under humid conditions in Puerto Rico, such that fallen berries may not serve as a major reservoir of the pest in this region [21,35]. Several studies have suggested that CBB can survive in alternate hosts (see Vega et al. [35] for a review); however, these claims are difficult to evaluate because CBB may be confused with other species of small scolytid beetles. In Puerto Rico, scolytid beetles were collected from possible alternate hosts and identified by DNA barcoding [35]. A small number of CBB were found in the fruits of *Inga vera* (a common shade tree on coffee farms) but there was no evidence of feeding or reproduction in fruits other than coffee, suggesting that alternate hosts are unlikely [35].

## 5. Coffee Growers’ Perception of IPM

When a new invasive insect pest is reported in a coffee-producing region, there is immediate concern among coffee growers, extension agents, and scientists. Growers hope for an easy, practical, and economically viable solution to control the new invasive pest. While the immediate response should be to focus efforts on the eradication of the new pest, this is often not possible since by the time the invasion is detected, it is often too widespread to eradicate. Management of the new pest is typically a more realistic scenario, and conventional growers’ first choice for control is often the application of pesticides. Unfortunately, control measures that rely solely on synthetic pesticides often fail due to the development of pest resistance to the chemicals, unintentional impacts on non-target organisms, and high costs of products and application.

The alternative to conventional management is to develop an IPM approach, which relies on a combination of control measures that are adapted to local coffee agroecosystems. However, the concept of IPM can be difficult to grasp, even for agricultural professionals and extension agents. IPM concepts can be even more challenging for coffee growers, who may not be well versed in the biology and management of pest organisms. Thus, when researchers and extension agents develop IPM recommendations for CBB, it is through farmer participatory research (FPR) that the adoption of IPM technologies is most likely to succeed. Examples of successful IPM programs for CBB that emphasize FPR are seen in Colombia, Ecuador, Honduras, and Mexico [36,37,38,39,40]. Through FPR, farmers are directly involved in the process of doing applied research to test the feasibility of IPM strategies that are adapted to their location [37,41,42]. Using FPR to “learn by doing” helps farmers select the techniques that work best for them, which facilitates the adoption of IPM strategies by additional farmers. FPR also helps farmers learn about new management techniques and concepts from their peers, in whom they may place greater trust than they do in extension agents or government employees.

Unfortunately, farmers’ perspectives on the control of insect pests and diseases are not always considered in the development of an IPM program. Researchers and extension agents may expect farmers to adopt IPM technologies without considering economic constraints or how this might impact their daily operations. Worldwide, 12.5 million smallholder farmers grow coffee on farms less than 5 ha and contribute ~60% of global production [43]. Despite most smallholder farmers having limited formal education, their empirical knowledge and understanding of local biotic and abiotic conditions are valuable when considering the development and implementation of IPM strategies. Their participation and ideas are relevant for testing new IPM alternatives, validating current strategies of control, disseminating knowledge among farmers, and improving IPM adoption. Research has shown that when coffee farmers are involved in field evaluations of CBB IPM practices, pest populations are successfully regulated, the quality of coffee is improved, the cost of CBB control is reduced, and the adoption of control strategies is increased [37,38,40,44].

## 6. Integrated Pest Management for CBB

An IPM program for CBB should include developing and validating pest monitoring plans, as well as cultural and biological controls. IPM programs must also consider the ecology, biology, and behavior of CBB and its relationship with coffee plant phenology under local environmental conditions. IPM components for controlling CBB have been developed in different coffee-producing countries such as Brazil [45,46,47], Colombia [9,10,23,48], Guatemala [49], and Mexico [11,50,51], with a focus on each specific coffee agroecosystem. While there are many other references from coffee-producing countries addressing each component of the IPM for CBB [19], the following sections focus on CBB IPM strategies that have been developed for Hawaii and Puerto Rico specifically and detail the challenges that persist for farmers, extension agents, and researchers.

## 7. Monitoring

**Figure 4:**
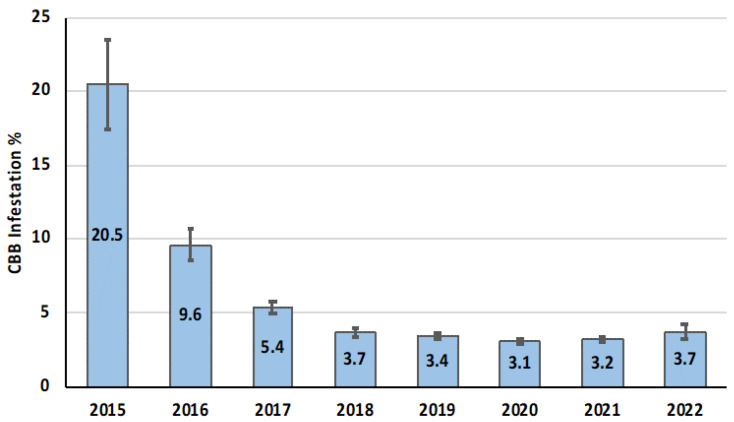
Significant reductions in CBB infestation (average per year) were observed from 2015–2022 in commercial coffee farms in the Kona and Ka’u districts of Hawaii Island because of implementing IPM strategies. Data from Aristizábal [54].

Monitoring CBB movement and infestation is one of the most important elements of an IPM program [19]. Monitoring provides critical insights into the population dynamics of CBB and provides coffee farmers with the baseline information needed to develop and implement effective IPM practices [19,52,53]. CBB populations are monitored using alcohol-baited traps and/or direct sampling of infested coffee berries [19,52,53]. Population monitoring informs the appropriate timing of management techniques such as spraying *B. bassiana* and can be used to assess how effective a given management strategy is. Monitoring also facilitates the detection of areas with high infestation levels (“hotspots”), which can then be targeted for management. The implementation of IPM strategies has resulted in a lower CBB infestation over time in the Kona and Ka’u districts of Hawaii Island (Figure 4).

### 7.1. Tree Sampling

In Hawaii, two strategies for monitoring CBB populations from infested berries have been refined and validated for statistical robustness. The 30-tree sampling plan for monitoring CBB infestation was developed in Colombia [9] and introduced to Hawaii in 2012 by Luis F. Aristizábal [55]. The 30-tree method is a good predictor of CBB populations even at a low rate (≤1%) of infestation [56]. This means that randomly selecting 30 trees in 1 ha (5000–10,000 trees) and counting all infested and uninfested berries on a single branch per tree (>45 berries ≥70 days old) is sufficient to obtain a realistic estimate of CBB infestation with 85% precision [56]. Although effective, researchers sought to improve on this sampling plan with the primary goal of reducing time inputs. The 30-tree sampling was modified and validated using a sequential sampling model on 17 commercial coffee farms on Hawaii Island [56]. Instead of counting the total number of green berries per branch plus infested green berries as indicated in the original 30-tree sampling plan, CBB infestation is estimated by counting only the infested green berries per branch on 24 trees, without a loss in precision or reliability (see Aristizábal et al. [56] for calculation details).

Pulakkatu-Thodi et al. [57] further validated this modified sampling plan using simulated actual precision. Their results showed that actual precision ranged from 70–80% using berry cluster sampling with the 75% precision plan and 88–92% for the 90% precision sampling plan. Sampling all fruits on branches returned very similar actual precision rates, suggesting that sampling clusters rather than whole branches from 30–35 trees was equally effective and labor-saving [57]. These precision levels held across a range of CBB infestation levels (0.5–12%). Using cluster sampling (3–5 clusters per branch), 25–53% fewer berries were counted with precision comparable to whole-branch sampling. This modified sampling plan is easier, more practical, and faster compared to the original 30-tree sampling.

In two separate studies, the spatial distribution (dispersion) of CBB within plantations was evaluated in Hawaii using Taylor’s Power Law. CBB has been observed to have an aggregated or clustered spatial distribution within Hawaii farms [56,57], as previously reported in Colombia [58]. This aggregated spatial distribution could be due to the limited dispersal distance of females from the berries where they emerged, the microclimate, or both. This distribution pattern means that CBB often form hotspots of infestation; therefore, control efforts should be intensified in those areas. The pattern of aggregated CBB infestation has implications for effective sampling. It is important to ensure that farms employ random sampling throughout a field to ensure that hotspots are not missed and that CBB infestation levels are not overestimated.

The degree of CBB penetration into the coffee berry is useful for determining the appropriate time to spray insecticides before damage has occurred. After applying an insecticide, the efficacy of the application can be determined by dissecting infested berries and counting the proportion of dead CBB females. The CBB female may be absent upon inspection of berries with an entrance hole; this may be indicative of mortality by *B. bassiana* or natural predators, or disturbance (e.g., during harvest). In Hawaii, spraying *B. bassiana* is recommended if the infestation is >3% with >25% of CBB in the AB position [56]. During the early coffee season (March–July) in Hawaii, a high proportion of CBB are in the AB position, which means the CBB females are vulnerable to insecticides such as *B. bassiana* or Pyronil [59,60]. Similar patterns were reported in Puerto Rico, with most CBB being found in the AB position early in the season (May–July) and in the CD position during the harvest and post-harvest (September–December) [61].

### 7.2. Traps

Alcohol-baited traps can be used to detect periods of high flight activity and can also be used to detect hotspots within fields and movement along borders [46,47,62,63]. Early detection of CBB movement within and between fields can aid growers in directing management activities to the areas where they are needed and at the appropriate time in the season, which may vary depending on the weather. The use of mass trapping during the post-harvest season has also been suggested as a method for controlling CBB [53], although only one study has attempted this [64].

In Hawaii, several studies have monitored CBB populations using funnel traps baited with a 3:1 methanol:ethanol lure [26,32,52,59,65]. Results of these studies showed peak flight activity in the early coffee season during berry development (February–May) and the late harvest season (November–January) on commercial coffee farms located in the Kona and Ka’u districts of Hawaii Island. Positive correlations were observed between CBB infestation levels and the number of CBB captured in traps [59], suggesting that the traps were an effective monitoring tool.

In Puerto Rico, homemade traps built from recycled 2 L soft drink bottles were common on farms for the first 5–10 years after the introduction of CBB. An average of 2000 to 7200 CBB/trap were caught per month, showing that the traps were efficient in capturing flying females [61]. Trends in CBB flight were like those observed in Hawaii, with peak flight activity early in the season during fruit development (April–June) and during the inter-harvest season (December–March) [61,66]. A recent study conducted in Puerto Rico reported a decreasing number of CBB captured as trap height increased [66], showing the preference of CBB to fly low to the ground (~0.5 m). In line with studies from Hawaii, the authors found a significant positive correlation between trap capture and berry infestation [65]. However, as was the case in Hawaii [30], coffee farmers in Puerto Rico gradually lost interest in the traps due to several factors, including the difficulty in obtaining ethanol and methanol for the lures as well as the time needed to check and maintain the traps. Additionally, some growers believed that the traps were attracting CBB from neighboring farms. After Hurricanes Irma and Maria in 2017, traps were no longer used on most farms in Puerto Rico.

**Figure 5:**
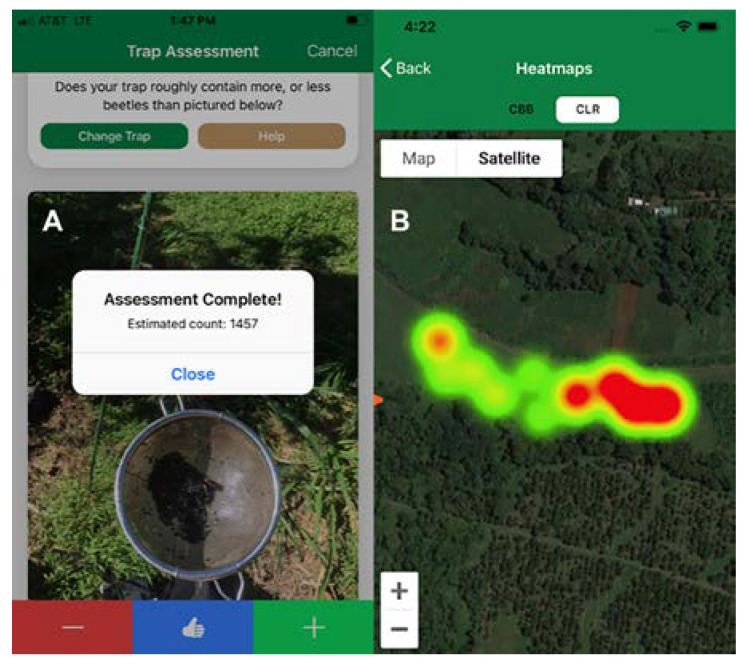
Best Beans app features: CBB trap count (**A**) and heatmap of infestation (warmer colors indicate high infestation) (**B**).

The observation of very few growers in Hawaii and Puerto Rico using the recommended methods of CBB monitoring led researchers at USDA-ARS to investigate the use of mobile application technology to reduce the time and effort needed to monitor CBB populations. The Best Beans app (available in the Apple Store and Google Play) employs a modified version of the 30-tree sampling method to monitor CBB infestation and position, as well as estimate the number of CBB caught in traps. The app uses geolocation information and provides the user with a map of the coffee field, including each coffee tree sampled, a heatmap of CBB infestation, and trap count estimates based on a curated library of thousands of photographs (Figure 5). In addition, Best Beans can be used to monitor coffee leaf rust (CLR, a recent invasive pathogen in Hawaii) infection. After trees are sampled for CBB and CLR, the app provides a summary of results and recommendations based on the collected data, as well as the time of year (flowering, berry development, or harvest season) and the weather conditions. Lastly, the app allows growers to log their management activities for accurate record keeping and sends text reminders to conduct relevant management practices throughout the year. The app has undergone testing on commercial coffee farms in Hawaii to improve its accuracy and practicality (Aristizábal and Johnson, unpub. data), and new features are currently under development.

## 8. Cultural Control

### 8.1. Harvesting and Sanitation Picks

Frequent and efficient harvesting, as well as post-harvest strip-picking (collection of all remaining green, ripe, and over-ripe berries at the end of the harvest season), are the most important cultural practices for regulating CBB as they remove population reservoirs from the fields [9,11,12,67]. While these practices are relatively simple in concept, their implementation is often limited by the cost of labor, field worker availability, and the quality of training they receive [9,10,29,39,68].

**Figure 6:**
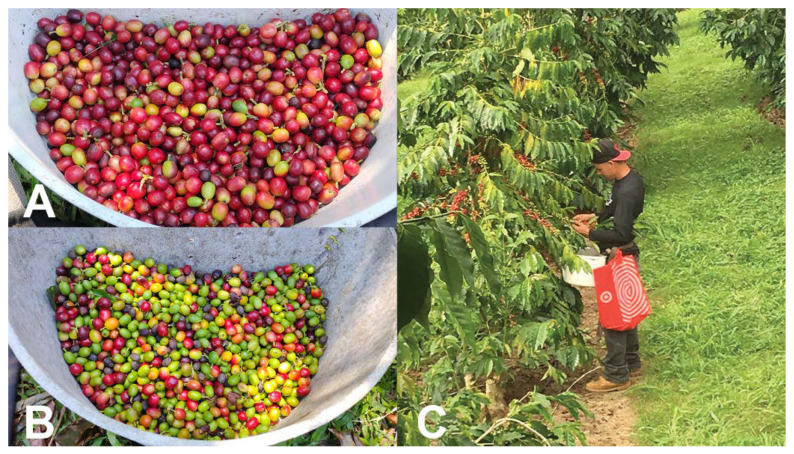
Standard harvesting and sanitation pick, in which only cherry and raisin berries are collected (**A**), strip-pick at the end of harvest season in which all berries are collected (green, cherry and raisin berries) (**B**), and a coffee picker working during the harvest (**C**). Photos: Luis F. Aristizábal.

In Hawaii, strip-picking is recommended as a critical part of the IPM program for CBB control [34,69]. This practice is regularly conducted in the Kona district of Hawaii Island on coffee farms that are at low and middle elevations (200–600 m), where the harvest season is short (3–4 months). In contrast, farms in the Ka’u district and at high elevations (>600 m) in the Kona district typically conduct sanitation picks (pre- or post-harvest removal of all ripe and over-ripe berries) since the harvest season is longer (6–9 months) in these locations [56]. The longer harvesting season is due to high precipitation in these locations, which allows multiple flowerings throughout the year. Flowers, developing green berries, and ripe and over-ripe berries are observed simultaneously during most of the year, making strip-picking economically unfeasible for coffee farmers in these locations. Instead of strip-picking, more frequent and efficient harvesting practices and sanitation picking (Figure 6) are recommended in areas that experience a year-round harvest [70].

The first evaluation of harvesting efficacy in Hawaii was conducted in 2016 on 11 coffee farms, with 36 harvesting rounds assessed [60]. The efficacy of each harvesting round was evaluated by randomly selecting 10 trees and counting the number of ripe and over-ripe berries left per tree (Excellent < 5 berries, Good = 5–10 berries, Bad > 10 berries [9]. Only 9.1% of rounds were scored as “Excellent”, while 21.2% were scored as “Good” and the remaining 69.7% were scored as “Bad” [60]. These results suggested that better training of coffee farmers and pickers was urgently needed to reduce CBB populations. In 2018, a second study evaluated 25 coffee farms in the Ka’u district. Prior to training coffee farmers and pickers in proper harvest techniques, there were an average of 15.6 cherries left per tree (“Bad”), and CBB infestation was 6.5% [54]. After farm visits and training were conducted, the average number of berries left per tree decreased to 6.3 (“Good”) and CBB infestation was reduced to 2.5% [54].

**Figure 7:**
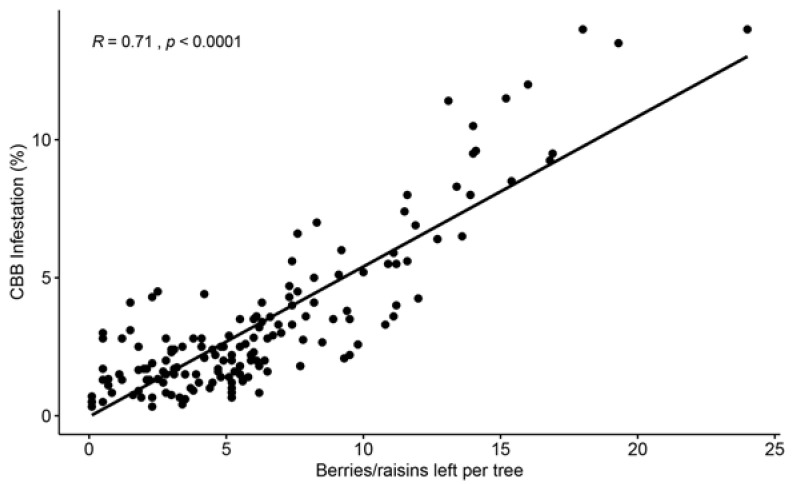
Positive correlation between the number of cherries and raisin berries left on trees after a harvesting round was conducted and CBB infestation. Evaluations were conducted on 10 commercial coffee farms from the Kona and Ka’u districts of Hawaii Island during the 2019–2020 coffee season (*n* = 184 evaluations). Data from Aristizábal et al. [70].

In a third study conducted on Hawaii Island, the efficacy of CBB control using conventional management (frequent sprays of insecticides and few harvesting rounds) was compared to cultural management (frequent and efficient harvesting and few insecticide sprays) over two years [70]. A positive relationship was found between CBB infestation, and the number of berries left per tree after a harvesting round (Figure 7). Frequent and efficient harvesting not only significantly reduced CBB populations and damage to processed coffee but was also found to be economically feasible for commercial coffee farms in Hawaii, which have some of the highest costs to produce coffee around the world. A cost–benefit analysis showed that a profit was obtained after sanitation, harvesting, and strip-picking were conducted, and the collected coffee was processed and sold [70].

### 8.2. Pruning

Pruning renews trees while helping to regulate CBB populations by allowing better access to berries during and after the harvest and improving spray coverage [9,19]. In Hawaii, the traditional pruning system is known as “Kona style”, in which each tree has multiple verticals of different ages [71]. In this pruning system, old verticals (>4 years old) with low production are removed, and only productive younger verticals are maintained [71]. This traditional pruning style promotes the growth of new verticals to improve the productivity and health of trees. However, it does not significantly reduce the CBB population since there is no interruption in berry development, which deprives CBB of food and shelter for reproduction and survival.

**Figure 8:**
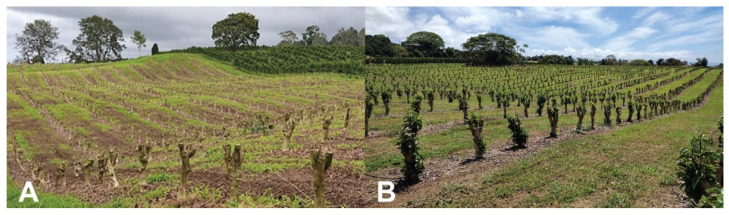
A coffee field block-stumped for the renewal of trees and control of CBB and CLR (**A**). Coffee field three months after stumping was conducted; note new leaf growth (**B**). Photos: Luis F. Aristizábal.

In contrast, stump pruning in blocks (Figure 8), initially developed in Colombia [9], guarantees a significant reduction in CBB populations in coffee lots since all verticals are removed and berry production is interrupted for 12–15 months. On stumped coffee lots, many CBB females emerge from berries fallen on the ground or left on stumped trees. CBB continues to emerge from these berries over a 3-month period [9,10] and can infest neighboring coffee lots. Keeping 1–2 rows of coffee trees along the border of stumped lots helps to capture flying females and limit the number that escape into neighboring fields [9,72]. These trap trees may be sprayed with *B. bassiana* and the berries collected every three weeks over the 3-month emergence period for the regulation of CBB [9,72]. Trap trees are then stumped to break the cycle. In Hawaii, stump pruning resulted in low CBB infestation (1–4%) in comparison to lots that used the Beaumont-Fukunaga pruning system (multiple verticals of the same age on each tree, with rows stumped in 3–5-year cycles), in which CBB infestation was 2–12% [60]. Additional studies are needed to fully evaluate the impacts of the various pruning systems on CBB populations as well as the cost and benefits of this cultural practice.

## 9. Biological Control

### 9.1. Beauveria Bassiana

The entomopathogenic fungus *Beauveria bassiana* (Basl. Criv.) Vuill. (Hypocreales: Cordycipitaceae) is a natural enemy of CBB in coffee-producing regions worldwide [9,11,12,15,73,74]. However, its efficacy as an inundative biological control agent is highly variable, with mortality ranging from 10–75% [9,10,48,75,76,77]. Weather conditions, virulence, pathogenicity, concentration, specificity of the *B. bassiana* strain, and formulation are some factors that determine the effectiveness of *B. bassiana* [4,78,79].

In 2011, the Hawaii Department of Agriculture (HDOA) authorized the distribution and application of commercial formulations of *B. bassiana* for the control of CBB. BotaniGard^®^ ES and Mycotrol^®^ ESO are two commercial formulations of the *B. bassiana* GHA strain (short for Grass Hopper Active, as it was originally formulated to control grasshoppers and locusts) that are widely used in Hawaii. The authorization of commercial formulations of *B. bassiana* marked the first step towards the establishment of an environmentally responsible IPM. Initially, coffee farmers conducted monthly calendar sprays of *B. bassiana* each season, which was costly in terms of products and labor and did not necessarily provide the level of control expected [60].

Later, the timing of applications was optimized based on trapping studies that elucidated peak CBB flight times [59,65], and susceptibility based on high percentages of CBB in the AB position [59,60,80]. Applications of *B. bassiana* became more effective and less costly since fewer sprays (4–5) were needed to obtain the same quality of coffee in comparison with calendar spray strategies (7–11 sprays per season) [79]. Cumulative mortality by the GHA strain on Hawaii coffee farms was estimated to be 20–60%, with sprays being most effective when conducted early in the season and under favorable weather conditions (overcast and humid, but not raining); half the recommended rate (16 Fl oz per acre) of BotaniGard^®^ ES was also reported to be as effective as full rates (32 Fl oz per acre) [81].

Several naturally occurring or “wild” strains of *B. bassiana* have been observed to infect CBB on Hawaii Island [80,82,83,84]. CBB infection and mortality induced by wild and commercial strains of *B. bassiana* were observed to increase with increasing elevation, emphasizing how microclimate influences the efficacy of this control [80,83]. Despite the initial optimism that wild strains of *B. bassiana* might offer the potential for improved CBB control in Hawaii, field [80] and laboratory studies [85] showed that the commercially formulated GHA strain was more virulent than wild strains, despite wild strains being more persistent in CBB populations [81].

In contrast, a recent study in Puerto Rico supported the potential benefits of wild *B. bassiana* strains. As in Hawaii, *B. bassiana* is often seen sporulating on CBB that have begun boring into coffee fruits in Puerto Rico and is often observed on farms where commercial strains have never been applied. Based on microsatellite DNA typing of numerous strains, the only commercially available strain in Puerto Rico (Mycotrol^®^) is genetically distinct from wild strains [86]. In lab experiments, the Mycotrol^®^ strain was very effective at killing CBB, and some wild strains were equally effective (though many were less virulent). When two wild strains (chosen for high virulence) were applied in the field, they were more effective than the Mycotrol^®^ strain in reducing the proportion of fruits with CBB damage and the number of CBB per fruit. Furthermore, the wild strains were recovered at a much higher rate than the Mycotrol^®^ strains, implying that they were more successful at surviving and reproducing under local field conditions after application [86]. This is not surprising considering that the Mycotrol^®^ strain was originally isolated in Oregon, USA, a very different climate than that of the coffee farms in Puerto Rico. However, US EPA regulations prohibit the use of strains and formulations not specified in product registrations. In other words, the registration of Mycotrol^®^ and Botanigard^®^ does not apply to other strains of the same species; the use of other strains would mean that the crop they are applied to cannot be sold legally in the US. The registration process would have to be repeated for each strain, a very expensive and lengthy process.

### 9.2. Flat-Bark Beetles

In Hawaii, two flat-bark beetles that persist as generalist predators and are commonly found in macadamia nut crops, *Cathartus quadricollis* (Coleoptera: Silvanidae) and *Leptophloeus* spp. (Coleoptera: Laemophloeidae), were found to feed on immature stages of CBB [87,88]. The flat bark beetle *Cathartus quadricollis* has also been found inside infested coffee fruits in Puerto Rico [89] and may play a small role in controlling the CBB population, especially in infested raisin berries left on trees [88,90,91]. To promote the increase in flat bark beetle predation on CBB, USDA-ARS researchers created predator breeding stations to distribute to coffee growers in Hawaii and augment existing populations. Studies to assess the contribution of these beetles to CBB mortality and the reduction in berry damage are ongoing (P. Follett, pers. comm.).

### 9.3. Ants

Several species of ants are predators of CBB larvae and pupae and can remove them from coffee berries [91,92,93]. In many cases, the ants nest inside the berries [93], while other species can predate or remove adult females before they begin boring into the endosperm [94]. Most of these reports are from observations in the field; relatively few experimental studies have tested the capacity of ants as predators of CBB. In Puerto Rico, the six most common species of ants on coffee farms are *Wasmannia auropunctata* (Roger), *Tapinoma melanocephalum* (Fabricius), *Monomorium floricola* (Jerdon), *Brachymyrmex heeri* (Forel), *Solenopsis invicta* (Buren), and *Paratrechina longicornis* (Latreille) [94,95]. The first four species are small ants, which could penetrate CBB entry holes in coffee berries and remove immature and adult CBBs. The other two species, *S. invicta* and *P. longicornis*, are larger species that could predate adult females before they reach and penetrate the berries. Of these six species, only *S. invicta*, *W. auropunctata*, and *Tapinoma* sp. significantly reduced the damage caused by CBB [94,96]. *Wasmannia auropunctata* (LFA, little fire ant) also significantly reduced CBB survival; this species has a great capacity to predate adult CBB inside coffee berries [94,97]. Its small size, high activity, and abundance in coffee plantations in Puerto Rico and Hawaii make LFA a candidate for biological control of the CBB. However, it is considered an agricultural pest due to its interference with harvesting and other field work; farm workers avoid areas with high abundance of this species due to its painful sting. *Solenopsis invicta* (RIFA, red imported fire ant) is larger than LFA but also potentially useful for the biological control of CBB in Puerto Rico. RIFA was observed to reduce CBB damage by predating adults before they reached the coffee berries [94]. Like LFA, its painful sting makes it extremely unpopular among orchard workers. Additional studies are needed in both Puerto Rico and Hawaii to fully characterize ant diversity on coffee farms and determine the level of CBB predation and removal from berries.

### 9.4. Parasitoids

**Figure 9:**
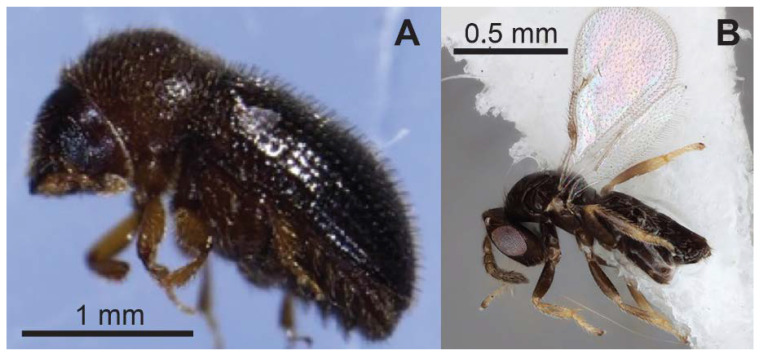
Coffee berry borer (**A**) and the parasitoid wasp *Phymastichus coffea* (**B**), a natural enemy of CBB that is slated to be released as a biocontrol in Hawaii. Photos: (**A**) M. A. Johnson, (**B**) David Honsberger.

Three species of parasitoid wasps have been identified as natural enemies of CBB in Africa: *Cephalonomia stephanoderis*, *Prorops nasuta* (Hymenoptera: Bethylidae), and *Phymastichus coffea* (Hymenoptera: Eulophidae) [98]. Of these, *C. stephanoderis* and *P. nasuta* can parasitize immature stages of CBB (larvae and pupae) and predate mainly small larvae and eggs [99,100,101], while *P. coffea* parasitizes adult CBB females [102,103] (Figure 9). Both *P. nasuta* and *C. stephanoderis* are also able to complete their life cycle inside the CBB-infested coffee berries [101,104]. Once inside the berry, female wasps paralyze or kill the CBB female and use its body as a barrier to limit the entry of other organisms [101]. Host feeding and oviposition take place after CBB females or bigger larvae and pupae are paralyzed [99,100,101,105]. Usually, the parasitoid attacks the CBB female through the dorsal part of the abdomen, laying two eggs [103].

Parasitism by *C. stephanoderis*, *P. nasuta*, and *P. coffea* tends to vary greatly from study to study, as summarized in Table 1. This variation is a disincentive to attempts to implement classical biocontrol programs. Competition and displacement between species of parasitoids have been observed. For example, competition for the host has been reported between *C. stephanoderis* and *P. nasuta*. *Cephalonomia stephanoderis* is more often successful, sometimes paralyzing and/or killing females of *P. nasuta* [106,107]. However, parasitoids can be used in tandem, with the release of *P. coffea* early in the season when coffee berries are developing, and most CBB females are in the AB position. This can be followed by the release of *C. stephanoderis*, which can parasitize and predate immature stages of the CBB [108].

In Puerto Rico, *C. stephanoderis* was imported in 2011 from CENICAFE in Colombia to quarantine facilities at the University of Puerto Rico in Mayagüez. However, delays in shipment meant that viable colonies could not be established. *Cephalonomia stephanoderis* has been observed to occur naturally on coffee farms in Puerto Rico; it was first reported in 2009, only two years after the first report of CBB on the island [89,127,128]. A viable colony established from individuals collected from coffee fruits in the field was established at the Agricultural Experimental Station in Adjuntas. In 2014, natural parasitism of CBB with *C. stephanoderis* was observed to reach 8%, and this increased to 20% following the release of lab-raised parasitoids [129]. Reductions in CBB-inflicted losses attributable to parasitoid impacts on beetle populations were estimated at 12–20% (USD 2.6–4.4M) at the farm level [129]. The parasitoid *P. coffea* was also imported to Puerto Rico from CENICAFE in 2010 and 2011. However, this species could not be released in the field because of requirements for host specificity tests and federal restrictions (F. Gallardo, pers. comm.). There are no reports of natural occurrences of *P. coffea* or *P. nasuta* in Puerto Rico. More detailed and extensive studies on the presence of these parasitoids in Puerto Rico are needed.

*Phymastichus coffea* is being considered for introduction into Hawaii as recent work has shown the high host specificity and capacity of these wasps to kill CBB females before they penetrate and damage the coffee endosperm [130]. Host specificity tests included 43 different species of Coleoptera, including non-target native Hawaiian species, exotic species, and beneficial species [130]. Results showed that only *H. hampei* and four other *Hypothenemus* species (*H. obscurus*, *H. seriatus*, *H. birmanus*, and *H. crudiae*) were parasitized [130]. Among the *Hypothenemus* species tested, those most distantly related to *H. hampei* were least parasitized, or not parasitized at all (*H. eruditis*) [130]. These results are promising, as high host specificity is among the most relevant aspects to consider for the introduction of biological control agents into Hawaii to ensure minimal risk for non-target native species. No native species of *Hypothenemus* occur in Hawaii; those that do occur are all invasive, and some are significant pests of other important crops such as Macadamia nut (*H. obscurus*) [130].

Yousuf et al. [130] suggest that the introduction of *P. coffea* as a biological control agent is highly likely to be environmentally safe. Now that permits have been obtained (May 2023) for importation and release, efforts will be made to establish *P. coffea* in Hawaii. Should establishment fail, *P. coffea* may be mass reared for inundative releases and incorporated into the current IPM program for CBB in Hawaii. Studies on rearing techniques, establishment, dispersal, impact on CBB populations, and compatibility with other CBB control strategies need to be addressed to facilitate the successful incorporation of this parasitoid into CBB management plans.

### 9.5. Entomopathogenic Nematodes

The potential use of entomopathogenic nematodes (EPNs), *Heterorhabditis* sp. and *Steinernema* sp., to control CBB has been reported in other coffee-producing countries [131,132,133,134]. In Hawaii, preliminary results showed the potential of *Steinernema carpocapsae* (Weiser) on infested green and raisin berries on the ground, in which CBB larvae mortality was 17.1% and 4.7% for adults [135]. In addition, two endemic EPNs from Hawaii (*S. feltiae* strain MG-14 and *Heterorhabditis indica* strain OM-160) tested on infested berries on the ground showed low mortality of CBB but high abandonment of CBB from infested berries [59]. Results suggest that there is potential for the use of EPNs against CBB, but additional field studies are needed to fully understand the role, effectiveness, and incorporation of those EPNs into an IPM for CBB.

### 9.6. Wolbachia Bacteria

One intriguing aspect of the CBB is its skewed sex ratio of approximately 10:1 females to males. A less skewed sex ratio would be advantageous because fewer female CBB would be available to attack coffee fruits. In many insects, skewed sex ratios are caused by infections of the endosymbiotic bacterium *Wolbachia*. The detection of *Wolbachia* in CBB Vega et al. [136] led Mariño et al. [137] to investigate its potential role in sex determination and reproduction in Puerto Rico. CBB colonies were fed artificial diets with the antibiotic tetracycline added to reduce populations of *Wolbachia*. After ten generations, *Wolbachia* was substantially reduced but not eliminated. The sex ratio was significantly less skewed than in controls, but not to the extent predicted. Thus, other factors appear to control the sex ratio in CBB. However, females on diets with tetracycline produced significantly fewer progeny, suggesting that reduction of *Wolbachia* (or other groups of bacteria; see Mariño et al. [138]) might affect CBB reproduction [137]. More detailed studies are needed to manipulate the *Wolbachia* infection of CBB for biological control. It is important to determine what mechanism causes the skewed sex ratio. Vega et al. [136] suggested that *Wolbachia* could induce cytoplasmic incompatibility in CBB, in which case the incompatible insect technique (IIT) [139] could be used. This technique involves mating populations of *Wolbachia*-free CBB females with infected males, and the resulting incompatible crosses would cause a decrease in CBB populations.

## 10. Chemical Control

The use of insecticides is intended to target CBB females when they are first colonizing and infesting new berries (AB position), before damage to the endosperm occurs. However, their effectiveness depends on timing sprays with CBB emergence, making good contact with the berries, applying them during favorable weather conditions, and proper calibration of sprayers. In many coffee-producing countries, synthetic insecticides containing highly toxic active ingredients (e.g., endosulfan, DDT, lindane, fenitrothion, fenthion, phenthoate, chlorpyrifos, and pirimiphos methyl-methyl) are the tools of choice used by farmers to control CBB [9,140,141]. In many cases, the use of insecticides is the first control strategy used by farmers since they are looking for a fast and effective solution. However, relying only on insecticides for control of CBB is not the best strategy since most of the CBB population is protected inside the berries and insecticides cannot reach them. In addition, their negative impact on human and environmental health, as well as the potential for CBB to develop resistance, has led to these chemicals being banned or phased out in several regions [53,140]. In Hawaii, relatively few products are authorized to be used in coffee to control CBB. A pyrethrin-based contact insecticide (Pyronil) has shown effective control of CBB [142], along with protectants such as Kaolin clay (Surround WP; Steiman and Burbano, unpub. data) and repellents (Verbenone; Wright et al., unpub. data). These products are applied alone or in a tank mixture with *B. bassiana* [69,79,142]. A recent field study testing the efficacy of spinetoram, whose active ingredient is derived from the fermentation of *Saccharopolyspora spinosa*, a naturally occurring soil organism, reported up to 73% control when CBB were in the AB position [143].

On Hawaii Island, several applications (4–5) of Pyronil or *B. bassiana* alone or in combination during the early coffee season (May, June, and July) were found to be as effective as monthly calendar sprays in controlling CBB but less costly [79]. However, sprays alone are often ineffective and must be combined with cultural control practices to achieve year-round control of this pest [79,81]. A viable economic strategy for controlling CBB in Hawaii includes a combination of monitoring, a few sprays of insecticides early in the season, frequent harvesting, and post-harvest sanitation [70]. Reducing the number of chemical sprays, and the use of less toxic insecticides, should be considered by farmers to preserve beneficial insects such as pollinators, predators, and parasitoids.

## 11. Agroecological Interactions

Most of the studies cited above have focused on a single method of controlling the CBB without considering other non-target organisms. These studies exclude the complex interactions between various organisms and trophic levels. For example, complete removal of remaining coffee fruits after harvest reduces the number of CBB that will survive to infest the next crop (F. Gallardo, pers. comm.), but also reduces the number of parasitoid wasps that attack them [122,123]. Pesticides applied to control the CBB also eliminate natural enemies of the coffee leaf miner (CLM), which can in turn become more destructive [144]. Interactions between pests and diseases of coffee and their natural enemies have been studied extensively for more than 25 years in Chiapas, Mexico (Perfecto and Vandermeer [145] and references cited therein). They describe a complex web of interactions at various trophic levels and suggest that, until this web is fully understood, control measures for one pest may have unintended consequences for others. For example, *Azteca* ants are predators of the CBB, but they also protect another pest of coffee, the green scale insect *Coccus viridis*. The scale insect can damage coffee plants, but it is also an alternative host for *Lecanicillium*, the hyperparasite of coffee leaf rust [145]. In Hawaii and Puerto Rico, there is no ant that is an apex predator, the equivalent of *Azteca* in Chiapas; the trophic web of each agroecosystem has its own cast of characters, many of which are still unknown. Since they are all interconnected, control measures that affect one of these organisms may have indirect effects on the others.

The potential introduction and release of *P. coffea* in Hawaii may be affected by sprays of *B. bassiana* and synthetic insecticides used for the control of CBB. Studies that focus on a single control measure for a single pest are oversimplifications and may miss important interactions with other organisms. Therefore, studies that address the compatibility among IPM strategies are needed to optimize their effectiveness and minimize collateral impacts on non-target organisms. However, it is not reasonable to expect that CBB management be delayed until the interactions between these two biological controls are fully understood, as this will likely take many years.

## 12. Costs and Benefits Associated with CBB Control

**Figure 10:**
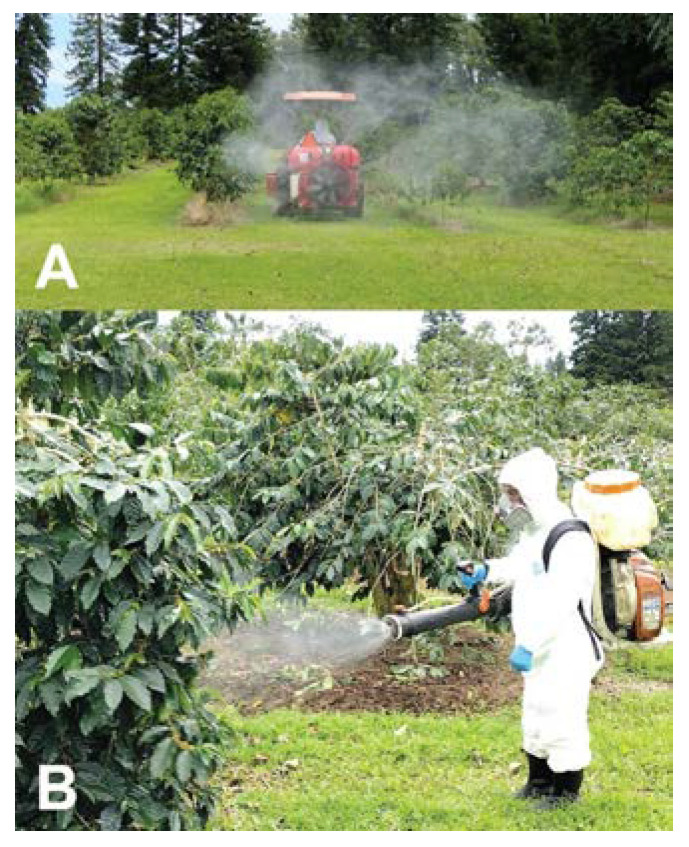
Spraying *B. bassiana* (BotaniGard^®^ ES) for CBB control using an air blast sprayer (100-gallon tank) mounted on a tractor (**A**) and a backpack mist sprayer (4-gallon tank) (**B**). Photos: Luis F. Aristizábal.

The cost of spraying pesticides to control CBB in Hawaii was estimated to be 5–12% of profits [79], a range very close to the 5–11% reported in Colombia [146]. A decision tree analysis examining pesticide sprays as the most important management decision during a crop season suggested that a low initial infestation at the start of the season was necessary to maximize net benefit [147]. This study found that the impact of the CBB subsidy program resulted in a net benefit of USD 947 for the average farmer. In a second analysis based on Hawaii data, Woodill et al. [147] found that all spraying strategies evaluated (always spray, IPM choice, or economic model) were more beneficial than not spraying, and that spraying was necessary to maintain profitability. The labor cost for spraying in Hawaii using backpack sprayers was reported to be USD 106–150 per spray (4–11% of profits), compared to USD 91–103 per spray (2–3% of profits) using air blast sprayers mounted on a tractor [148] (Figure 10). The slope and terrain of most Hawaii farms, particularly in the Kona district, prevent the use of tractor sprayers and thereby limit the ability of growers to apply pesticides.

For these farms, cultural controls may be the best alternative to chemical sprays, which are costly in terms of products and labor. Frequent harvesting and proper timing of several *B. bassiana* sprays early in the season based on monitoring results were determined to be the most effective and least costly CBB control strategy on commercial farms in Hawaii [70]. A focus on cultural control practices and 0–3 sprays per season resulted in a 48% increase in net profits and a 55% decrease in the cost to control CBB using chemicals, relative to conventional strategies that relied on calendar sprays (4–7 per season) and few harvesting rounds [70].

The total economic benefit of CBB management in Hawaii between 2011 and 2021 was estimated to be USD 251M [16]. The use of *B. bassiana* alone resulted in an economic benefit of USD 52M, while the early adoption of IPM strategies by farmers represented an economic benefit of USD 69M. However, the highest economic benefit (USD 130M) was obtained after the IPM recommendations were supported by Hawaii-based research [16]. This emphasizes the value of continued research on this economically important pest.

## 13. Current Status of CBB IPM in Puerto Rico and Hawaii

In the first decade of CBB presence in Puerto Rico (2007–2017), several control measures were commonly implemented. The University of Puerto Rico’s Agricultural Experiment Stations taught coffee farmers how to make alcohol-based traps and encouraged their implementation [149]. The Puerto Rico Department of Agriculture subsidized the use of Mycotrol^®^ (a biopesticide containing the entomopathogenic fungus *Beauveria bassiana*) to control CBB and applied it on many coffee farms. The University of Puerto Rico Mayagüez and Casa Pueblo, a visionary community organization in the coffee-producing town of Adjuntas, taught farmers how to grow and apply their own *B. bassiana* [150]. Processing centers (called beneficiadores) that buy coffee penalized sellers whose coffee had high levels of infestation, using a scale prepared by the Agricultural Experiment Station [151]. However, as time passed and CBB infestation was low in some years, farmers grew complacent about CBB control and shifted their focus to other problems such as coffee leaf rust (CLR) and coffee leaf miner (CLM), both of which affect leaf longevity and berry production, and can often be worse than CBB. Fewer farmers used traps (old, abandoned traps are still commonly seen on coffee farms) and applied *B. bassiana*. Processors stopped paying less for coffee with extensive CBB damage, which lowered the quality of the crop. After Hurricanes Irma and Maria devastated coffee farms and infrastructure in 2017, even less attention was paid to CBB control. Farmers were trying to reconstruct infrastructure and replace coffee plants that had been destroyed. CBB control faded from their collective consciousness. Today, most farms in Puerto Rico apply no control measures at all.

After more than a decade of living with CBB in Hawaii, some similarities and differences with Puerto Rico can be seen. As in Puerto Rico, very few growers in Hawaii currently use traps or tree monitoring to inform spray decisions. Instead, growers may either visually inspect a small number of berries for CBB presence to inform spray times or are on a calendar spray system whereby fields are sprayed monthly regardless of infestation levels. Subsidies for *B. bassiana* are still available to growers in Hawaii, but only a small percentage of the total farms in the State take advantage of this program. At the core of these issues is a combination of an aging farmer population, a disconnect between farm owners and the issues that are occurring on their fields, and high costs that prohibit many growers from implementing the management that they know is needed but which they cannot afford. Few growers are willing to conduct monthly monitoring due to the time and physical effort required. Many of the management decisions that take place on these farms are left to field managers or seasonal workers who may have little knowledge of practices that have been optimized for Hawaii. This is often due to a combination of language barriers (most farm workers are Latin American, Micronesian, or Filipino), and a lack of communication between farm owners and workers. Additionally, many workers have coffee experience outside of Hawaii and often believe that practices that are effective in their home countries will be equally effective in Hawaii, and this is unfortunately not always the case. Lastly, the emergence of CLR in Hawaii in late 2020 has forced growers to shift their focus to managing this crippling disease that has reduced yields by up to 75% in Kona during the 2022 harvest season. Many growers with small farms and limited profits must now decide whether to apply *B. bassiana* or fungicides if they cannot afford both. Confusion also exists in understanding the compatibility of fungicides, *B. bassiana*, and other inputs such as foliar fertilizers. Some growers suffered heavy production losses when they combined products that were incompatible in the 2022 season, emphasizing the need for research that determines product compatibility under field conditions.

## 14. Conclusions

The development and implementation of an IPM program for CBB is not an easy task, but achieving efficient, economical, and environmentally friendly management of this pest in Hawaiian and Puerto Rican coffee agroecosystems is feasible. Three key aspects for establishing a successful IPM program against CBB include understanding the biology and ecology of the pest, monitoring fields regularly to inform spray decisions, and combining several management techniques to achieve comprehensive year-round control. First, understanding the biology, ecology, and behavior of CBB in local coffee agroecosystems and its relationship with coffee plant phenology, weather, and natural enemies allows the estimation of development times, dispersal patterns, survival, and the impact of environmental factors on the CBB population. Second, monitoring CBB populations can aid in detecting periods of elevated flight activity and identifying hotspots within fields, both of which are relevant for timing and targeting sprays of insecticides or *B. bassiana*. Third, the combination of two or more management practices such as cultural controls (pruning, efficient harvesting, and strip-picks) and sprays of *B. bassiana* early in the season has been shown to be more efficient and cost effective than single practices alone. Biological control methods, including the promotion of natural enemies (flat bark beetles, ants) and the introduction of parasitoids (*P. coffea*), are aspects that need to be evaluated for efficacy and incorporation into IPM programs. Lastly, the participation of coffee farmers in the basics of applied research is vital to the successful control of this pest and is perhaps the most relevant aspect to consider as they must ultimately decide which IPM strategies are going to be applied and established on their farms.

From 2007–2023, 53 peer-reviewed articles, chapters, or books were published on CBB in Puerto Rico and Hawaii (Table 2). The majority of these documented the use and optimization of *B. bassiana* (15%), various aspects of CBB biology and ecology (15%), and IPM strategies (12%). Poorly studied areas include potential biological controls (e.g., parasitoids), physical controls, and chemical controls (Table 2). More research has been conducted on CBB in Hawaii (40 publications) compared to Puerto Rico (13 publications); while Hawaii-based research has been broad in nature, research in Puerto Rico has been focused on biological control and CBB biology. No articles have been published on precision agriculture tools in either region. Potentially promising areas of research include drone spraying, mechanical harvesting, and mobile applications, all of which could decrease labor and production costs and deserve further attention as strategies that could be used in combination with other IPM techniques.

Finally, more diversified methods of outreach and education are needed to reach the wide variety of growers across Hawaii and Puerto Rico. Significant gaps exist in the ability and willingness of growers and workers to access and digest information online, emphasizing the importance of on-farm workshops and farmer-to-farmer teaching. Additional methods of training are needed to help coffee farmers and field workers learn how to properly conduct cultural controls and optimize the use of biological control agents such as *B. bassiana*. Although considerable effort has gone into the development and implementation of CBB IPM for Hawaii and Puerto Rico’s coffee agroecosystems, the adoption of these strategies by coffee farmers needs to be increased.

## Figures and Tables

**Table 1:** Summary of studies using the parasitoid wasps *Cephalonomia stephanoderis, Prorops nasuta*, and *Phymastichus coffea* for biological control of the coffee berry borer (CBB).

Parasitism Type	Parasitoid	Country	CBB Parasitized (%)	Result	References
Natural	*C. stephanoderis*	Brazil	0.5–83	Not reported	[109]
Natural	*C. stephanoderis*	Brazil	2–24	Not reported	[110]
Previous Release	*C. stephanoderis*	Mexico	0.3–26	Not reported	[51,111]
Natural	*P. nasuta*	Brazil	2–33	Not reported	[112]
Previous Release	*P. nasuta*	Colombia	0.25–50	Not reported	[113,114]
Release (1–3)	*C. stephanoderis*	Colombia	2–8 25–65	CBB mortality 95% CBB adult predation 94%	[115] *, [116,117]
Release (1)	*C. stephanoderis*	Guatemala	5–91	Reduction in infestation (1–15%). Reduction in CBB population (2–43.1%)	[118] *
Release (1)	*C. stephanoderis*	Ecuador	3–52	Not reported	[119]
Release (1)	*C. stephanoderis*	Colombia	Not reported	Reduction in CBB population (43–73%)	[105] *
Release	*C. stephanoderis*	Colombia	Not reported	Reduction in infestation (11%)	[120] *
Release (3)	*P. nasuta*	Colombia	1.5–44	Reduction in infestation (46% on average)	[117] *, [121] *
Release (1)	*P. nasuta*	Ecuador	0.3–22	Not reported	[119]
Release	*P. coffea*	Colombia	2–95	Reduction in infestation (47%)	[120] *, [121,122,123,124,125]
Release	*P. coffea*	Mexico	10–97	Reduction in CBB infestation (2–81%) Reduction in CBB population (90–96%)	[103] *, [126] *
Release	*P. coffea*	Colombia	Not reported	Reduction in CBB infestation (47%)	[120] *
Release	*P. coffea*	Brazil	Not reported	Reduction in CBB infestation (18%)	[120] *

* In these studies, the effect of parasitoid releases on number of CBB and/or CBB infestation was reported. Numbers in parentheses indicate number of releases of parasitoids in field.

**Table 2:** Peer reviewed publications (articles, chapters, books) on coffee berry borer (CBB) in Hawaii and Puerto Rico from 2007–2023.

Topic/Sub-Topic	Publications	References
**CBB detection and dispersal**	4	[18,25,27,28]
**CBB biology and ecology**	8	[31,32,34,35,61,65,152,153]
**Integrated pest management**	6	[19,29,32,55,56,60,69]
**Trapping**	3	[26,59,66]
**Monitoring**	4	[21,52,57,142]
**Cultural control**	3	[54,70,154]
**Biological control**		
*B. bassiana*	8	[79,80,81,82,83,84,85,86,155]
Flat bark beetle	3	[87,88,90]
Ants	2	[94,95]
Parasitoids	1	[130]
*Bacillus*	1	[156]
*Wolbachia*	1	[137,138]
Nematodes	1	[135]
**Physical control**	1	[20]
**Post-harvest**	1	[157]
**Chemical control**	1	[143]
**Economics**	4	[16,147,158,159]
**Total**	**53**	

## Data Availability

Not applicable.
